# A Harris Hawk Optimisation system for energy and resource efficient virtual machine placement in cloud data centers

**DOI:** 10.1371/journal.pone.0289156

**Published:** 2023-08-11

**Authors:** Madhusudhan H. S., Satish Kumar T., Punit Gupta, Gavin McArdle

**Affiliations:** 1 Department of Computer Science & Engineering, Vidyavardhaka College of Engineering, Mysuru, Karnataka, India; 2 Department of Computer Science & Engineering, BMS Institute of Technology & Management, Bengaluru, Karnataka, India; 3 School of Computer Science, University College Dublin, Dublin, Ireland; Nottingham Trent University School of Science and Technology, UNITED KINGDOM

## Abstract

Virtualisation is a major technology in cloud computing for optimising the cloud data centre’s power usage. In the current scenario, most of the services are migrated to the cloud, putting more load on the cloud data centres. As a result, the data center’s size expands resulting in increased energy usage. To address this problem, a resource allocation optimisation method that is both efficient and effective is necessary. The optimal utilisation of cloud infrastructure and optimisation algorithms plays a vital role. The cloud resources rely on the allocation policy of the virtual machine on cloud resources. A virtual machine placement technique, based on the Harris Hawk Optimisation (HHO) model for the cloud data centre is presented in this paper. The proposed HHO model aims to find the best place for virtual machines on suitable hosts with the least load and power consumption. PlanetLab’s real-time workload traces are used for performance evaluation with existing PSO (Particle Swarm Optimisation) and PABFD (Best Fit Decreasing). The performance evaluation of the proposed method is done using power consumption, SLA, CPU utilisation, RAM utilisation, Execution time (ms) and the number of VM migrations. The performance evaluation is done using two simulation scenarios with scaling workload in scenario 1 and increasing resources for the virtual machine to study the performance in underloaded and overloaded conditions. Experimental results show that the proposed HHO algorithm improved execution time(ms) by 4%, had a 27% reduction in power consumption, a 16% reduction in SLA violation and an increase in resource utilisation by 17%. The HHO algorithm is also effective in handling dynamic and uncertain environments, making it suitable for real-world cloud infrastructures.

## Introduction

Cloud computing is a paradigm for providing on-demand computational services and resources through the internet, such as data storage and computing power [1]. Cloud computing offers customers on-demand resources in the form of virtual machines (VMs) and accomplishes their tasks while meeting Quality of Service (QoS) requirements. Each VM is designed to target a certain computing resource capability (e.g., the number of CPUs, I/O bandwidth and memory capacity). Using a physical machine (PM) or host to run several VMs, Virtualisation technology increases a data centre’s energy efficiency by decreasing the amount of hardware in use and increasing the resource usage of physical machines. Cloud providers need to schedule the virtual machines to suitable physical machines so that both users’ and the providers’ objectives will be optimised.

The notion of cloud data centres comes from the fact that cloud computing makes use of data centre infrastructure to offer services. Cloud data centres will manage 94% of workloads by 2021 [2]. However, the operations of these data centres require a lot of energy. Energy expenses account for about 42% of total operational costs, according to Amazon’s data centre research [2]. Another reason to save energy is the ongoing discussion about climate change. Running servers is projected to produce 0.5% of world CO_2_ emissions [3]. As a result, lowering data centre energy usage without sacrificing the QoS provided is an incipient research domain.

Large numbers of physical servers are commonly seen in data centres. The IT infrastructure, which is subjugated by PMs, accounts for about 60% of the overall energy usage in a data centre. Virtualisation is a technique that allows customers to access cloud computing resources in the form of several VMs. Since numerous VMs may be deployed to a similar physical server, Virtualisation is critical for attaining both energy efficiency and high server utilisation. Hence, employing an effective Virtual Machine Placement (VMP) method can have a significant impact on the power usage of a data centre. VMP is an NP-hard optimisation problem [4].

Virtual machines (VMs) share resources through Virtualisation on hosts to process user requests over physical machines (PMs). Virtualisation may be used to conduct three different operations: VM migration, VM isolation and VM consolidation. The virtual machine migration technology moves virtual machines from one PM to another. Virtual machines operating on separate hosts will leave that host and congregate on fewer ones during the VM consolidation process to save energy by turning off or transferring the initial running host to hibernate mode [5].

The issue of energy consumption has improved because of recent developments in hardware technology. It is still a major issue for sustainable computing though, because how computing and auxiliary hardware resources are used has a significant impact on how much energy is used by those resources. In contrast to resources that are employed effectively, underutilisation or over-utilisation of the resources (CPU and RAM) results in increased energy consumption. This necessitates the creation of several software energy-saving strategies, such as scheduling and Virtualisation. With lower resource utilisation, the energy efficiency of the system will be lower. Additionally, there will be more active hosts.

A PM supplies all essential VM resources such as storage, network bandwidth, memory, and CPU as each PM can hold several VMs. Consolidation of virtual machines is a method for making intelligent and efficient use of the resources of the cloud. One of the most difficult components of VM consolidation is VM allocation. It is described as locating the best PM for a VM to decrease the number of operating physical machines in data centres. As a result, many goals have been proposed for improving load balancing, lowering costs and network usage, mitigating SLA (Service Level Agreement) violations, increasing energy efficiency, and maximising resource utilisation.

**Fig 1:**
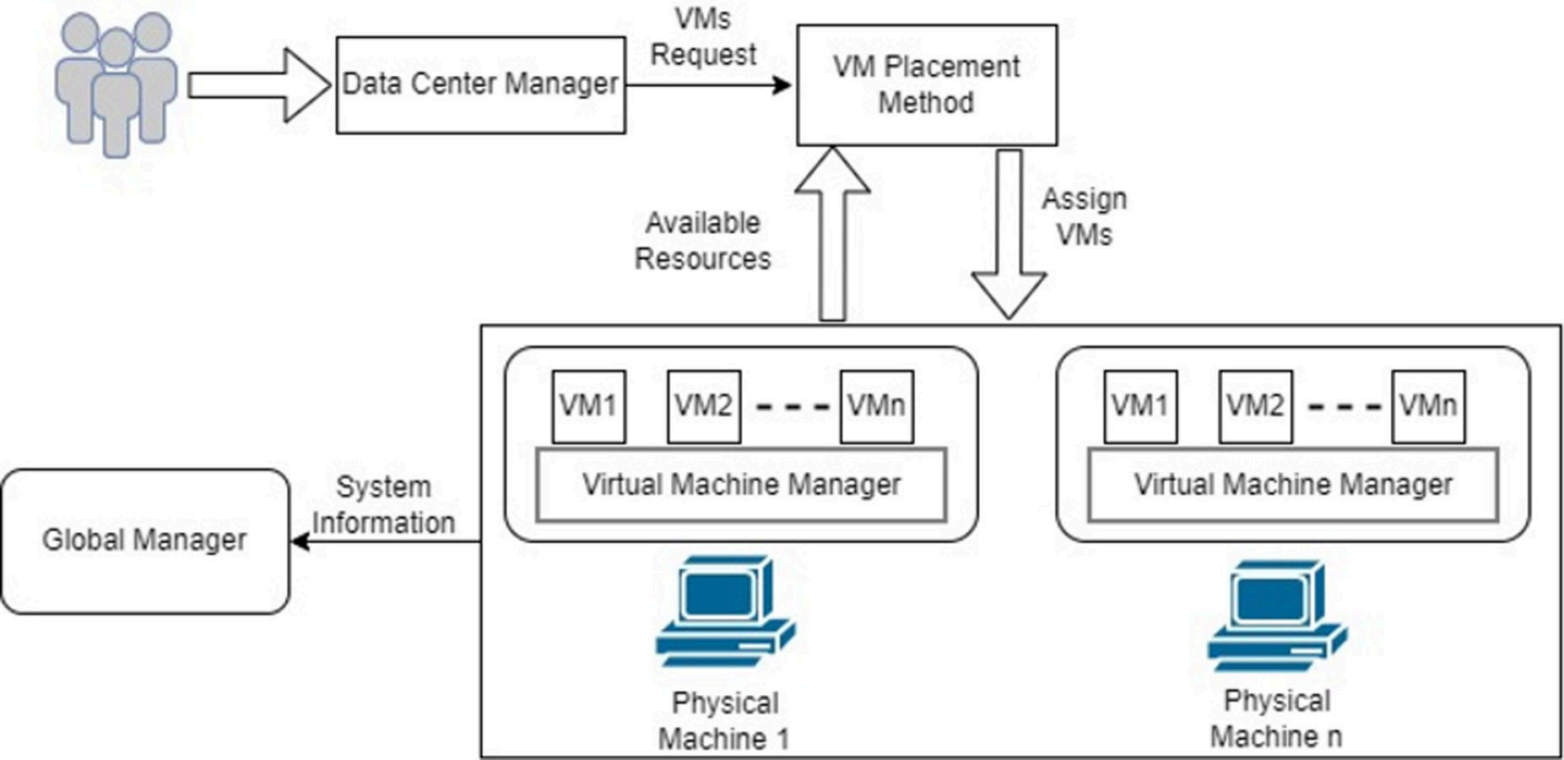
System design for VMP in the cloud.

This work presents a Harris Hawk Optimisation (HHO) model for multi-objective virtual machine placement in the cloud data centre. The proposed HHO model aims to optimally place VMs on appropriate physical hosts. HHO is a meta-heuristic approach for determining the global ideal solution. The system model is depicted in Fig 1. A data centre is made up of multiple physical machines. Many virtual machines can run on a single physical machine. Virtual Machine Manager (VMM) also identified as Hypervisor, is software that makes it easier to create, manage, and monitor virtual machines. On top of physical hosts, it also controls a Virtualised environment. When the data center manager receives a request for VM execution it first gathers status information from all accessible physical machines and sends it to the VM scheduler. The HHO model was used to create the VM scheduler. The VM scheduler then evaluates the status information and assigns VMs to appropriate PMs.

Harris Hawk is a meta-heuristic approach for determining the global ideal solution. The significant contributions of the proposed work are listed below:

- A resource and energy-efficient VM deployment model for diverse cloud environments is proposed. This contribution intends to increase the resource utilisation and then the energy consumption can be minimised while satisfying the QoS expressed by the cloud providers.
- Load balancing is addressed by migrating VMs from overloaded to underloaded physical machines and vice versa.
- Reducing the running time of the VM Placement algorithm: The reduction in execution time required to process all the requests from users plays a vital role from the cloud provider’s perspective since it directly affects the performance of the cloud provider.

The remainder of this article is structured as follows, a review of the existing techniques is presented in the literature review section. The problem formulation section discusses the problem and the proposed HHO model, while the experimental setup and comparisons section evaluates the proposed technique. Finally, the conclusion section summarises the paper with a discussion on future work.

## Literature review

One of the challenges with cloud computing is VMP which has an impact on many aspects of cloud computing. As a result, several research efforts have been carried out to map the best position for VMs among accessible PMs. This section summarises pertinent studies on VMP and Table 1 depicts the studies and their parameters. Also, virtual machine placement technquies are categorized into Nature/Bio Inspired methods, Metaheuristic approaches and Machine Learning technquies.

**Table 1:** Classification of different approaches in the existing literature.

Reference	Algorithm Used	Parameters				Limitations
		Execution Time	Resource Utilisation	Energy Consumption	SLA Violation	
6	Salp Optimisation			✓	✓	Doesnot consider Resource Utilisation
7	Whale optimisation algorithm					Focused only on bandwidth efficiency, does not consider resource utilisation and energy consumption
8	PSO			✓		Doesnot consider resource utilisation, execution time and SLA violations.
9	ACO	✓		✓		No real-time dataset is used, and resource utilisation is not considered
10	Proportionate resource utilisation (PRU) based policy		✓	✓		Does not consider execution time and SLA violations
11	Dolphin partner optimisation		✓	✓	✓	Does not consider VM migrations
13	Original harmony search			✓	✓	Does not consider resource utilisation and execution time
14	Whale optimisation		✓	✓		Randomly generated data were used and does not consider SLA violation
15	HPSOLF-FPO		✓	✓		Randomly generated data were used and does not consider load balancing
18	ACLR			✓		Focused only on energy consumption
20	Firefly algorithm	✓				Resource and energy optimization was not considered
22	Q Learning			✓		Focused only on energy consumption
23	Flower pollination	✓	✓	✓		SLA violation is not considered
29	Artificial ant colony	✓	✓			The work aims to improve only Makespan and resource utilisation
30	Self-adaptive PSO	✓				The work aims to improve only the cost and execution time of the task.
31	Multi-agent system	✓				Focused on execution time and parallel resource utilisation. Energy consumotion is not considered
32	Double deep Q-network			✓		Only to improve power and network load
33	Flower Pollination Algorithm		✓	✓		Improve power efficiency of host and number of migration. Does not consider resource utilization
34	krill herd			✓	✓	Improves only power consumption and minimize SLA violation. Resource utilization is not considered.
35	Deep reinforcement learning algorithm	✓		✓		Does not cosider resource utilization and SLA violation
36	Ant lion optimizer and sine cosine algorithm		✓	✓	✓	Does not consider execution time
38	Symbiotic Organisms Search Algorithm		✓	✓		Doesnot consider execution time and SLA violation
39	Enahnced Cuckoo search algorithm			✓	✓	Does not consider resource utilization
40	Squirrel search algorithm	✓	✓	✓	✓	Migration cost is not taken into consideration
41	Clonal optimization	✓	✓			SLA and resource utilization is not taken into consideration
42	Hybrid BAT and ABC	✓				Doesnot consider Resource Utilisation and energy optimization
43	Jelly Fish	✓	✓			Energy and SLA is not considered in this work
46	Elephand herd	✓		✓		SLA and migration cost is not taken into consideration
47	Whale	✓	✓	✓		SLA and migration cost is not taken into consideration
48	Ant Lion	✓		✓		Resource utilization and SLA is not considered
49	Buterfly optimization	✓	✓			Energy and SLA is not considered in this work
50	Gray Wolf	✓	✓	✓		SLA and migration cost is not taken into consideration

### Nature/bio inspired methods

The Salp Swarm Algorithm and the sine-cosine algorithm were combined to create a hybrid multiobjective VM placement technique [6]. The proposed technique aimed to reduce the mean time before host shutdown (MTBHS), the number of SLA violations and power consumption. The proposed method was compared to several meta-heuristics, and the findings showed that it was superior. When discussing VMs and PMs, the bandwidth has not been fully considered. Furthermore, the balanced use of multidimensional resources in physical hosts remains uncertain.

A bandwidth-aware VMP algorithm, developed on the enhanced Whale Optimisation Algorithm (WOA) and a novel bandwidth allocation methodology, was proposed in [7]. The outcomes reveal that the suggested method outperforms several meta-heuristics and heuristics. I suggested approach focuses solely on bandwidth optimisation, neglecting to consider other critical resources like memory and CPU use. Also, the study did not focus on the problem of optimising power consumption.

The authors in [8] proposed an energy-aware VM placement technique using Binary Particle Swarm Optimisation (BPSO) algorithm. The work is based on the modification of local optimum placement and global optimal placement, to get optimal VM placement with the lowest energy usage.

To minimise energy consumption and fulfil uers’ experience, an enhanced ant colony algorithm is used to propose an energy-saving VMP approach which attains a balance between user experience and energy consumption in data centres [9]. The original ant colony algorithm’s pheromone and heuristic parameters were modified, ensuring that the improved algorithm may transition from a local to a globally optimal solution, avoiding the algorithm’s early maturity. Dolphin Partner Optimisation along with optimised security for resource allocation, has been presented in [10]. Memory-aware Optimisation and Energy-based Prioritisation are utilised to pick memory and energy-established VMs for safety, and this work has also incorporated hypervisor safety into the two groups of VMs acquired. The Dolphin Partner Optimisation then enhances the two sets of virtual machines to provide the best capable VM for each set. Finally, streamlining security is applied to boost security, and the chosen virtual machine is essentially the most secure.

Authors in [11] proposed a research model that uses VM consolidation to minimise data centre power consumption while maintaining stable operation. An adaptive harmony search approach is created to achieve the best solution for the suggested VM consolidation model, which requires less effort to establish the model’s parameters than current harmony search methods.

The authors in [12] presented an energy-efficient container placement using the WOA technique. Two stages of placement, that is placing containers on suitable VMs and mapping VMs to suitable PMs are solved as one optimisation problem. The proposed method is evaluated in a heterogeneous environment and results show, it minimises the power consumption, reduces the number of PMs used and maximises the resource utilisation but increases the number of migration increasing the cost.

Authors [13] developed a hybrid approach using PSO and Flower Pollination Optimisation techniques to reduce power consumption, placement time, and resource wastage and increase server utilisation. Placements of the virtual machines onto physical machines are accomplished based on the fitness values derived from the above objectives.

Adlin Sheeba et al. proposed a VM placement technique using the Firefly Optimisation Technique [14]. In this work, the authors used the K-Means clustering technique to minimise the migration time of VMs. An enhanced Firefly Optimisation Algorithm was used to design the VMP model. To decide the optimal cluster for VMP, a combination of PSO and coyote algorithm was used.

In [15], the authors proposed a Water Wave Optimisation technique to handle virtual machine consolidation problems in the cloud. The proposed method is employed to find the proper migration plan to minimise the load on the overloaded hosts and maximise resource utilisation. In [16], a Flower Pollination-Based Nondominated Sorting Optimisation (FP-NSO) algorithm is presented to handle VM placement to minimise energy consumption and maximise resource utilisation. The method that aids in identifying the most suitable PMs for deploying VMs in a cloud environment is linked to many resource-constraint parameters.

In a recent work [17], the author has proposed a modified ant colony-based load balancing algorithm for cloud resource optimisation to improve makespan and resource utilisation in the cloud. Similar work using self-adaptive PSO (Particle Swarm Optimisation) [18] is proposed to improve cost using a combination of machine learning to predict the cost model and PSO for finding the best resource over the cloud. The work aims to improve the cost of the resources and execution time. In [19], a resource allocation algorithm is proposed using The Flower Pollination Algorithm to improve power efficiency as compared to a genetic algorithm in the cloud. This work also tried to reduce the number of migrations to improve resource utilisation (CPU and RAM utilisation). From the nature-inspired algorithms, the krill herd model has been proposed to improve SLA violation and energy efficiency in the cloud [20]. The work shows an improvement in SLA and power efficiency as compared to the genetic algorithm and the MBFD (Modified Best Fit Decreasing) algorithm. Authors in [21] proposed a hybrid approach for multi objective virtual machine placement in cloud. Ant lion optimization and sine cosine algorithm were used to optimally place VMs over suitable physical machines. Performance metrics like power consumption, resource wastage, reosuce utilization, number of active PMs, VM migrations and SLA were considered.

In [22], authors presented a Variable Neighborhood Search-Based Symbiotic Organisms Search Algorithm to enhance energy efficiency in cloud. Authors aimed to minimize energy consumption and maximize resource utilization. A minimum of active hosts and the energy-saving turnoff of inactive servers allowed for the best VM allocation. Esha Barlaskar et al., [23] proposed an enhanced cuckko search algorithm to obtain optimal solution for virtual machine placement in cloud. This work aims to reduce energy consumption, VM migrations and SLA violation. Hetergeonous hosts were used for experimentation work. In [24] authors has proposed an nature inspired squirel search optimization algorithm for resource scheduling is cloud. the work is compared with genetic algorithm, PSO and ACO using energy, cost and SLA as performance parametrs. In [25] a clonal optimization model is proposed for resource allocation for cloud infrastructure to improve power efficiency in cloud. In recent years many other work are been proposed using nature inspired algorithms like work inspired from BAT algorithm [26], jelly fish [27], wild horse [28], FOX inspired model [29], Elephant herd [30], Whale Optimization Algorithm (WOA) [31], Ant Lion [32], Butterfly Optimization Algorithm (BOA) [33] and Gray Wolf Optimization (GWO) [34].

### Metaheuristic approaches

A VM allocation policy has been proposed that assigns VMs to hosts proportionally based on their RAM and CPU use. It employs the idea of skewness to assess the unevenness in host resource utilisation and assigns VMs to the host machine with the lowest skew value [35].

In [36], a combination of a mixed integer linear program and a heuristic approach was proposed for virtual machine placements in edge-cloud computing. The objective is to meet the varied latency requirements of applications while minimising the consumption of IT infrastructures for the placement of VMs in cooperative edge-cloud computing. To defend against co-location assaults in IaaS (Infrastructure As A Service) cloud providers, the authors in [37] presented a VM allocation strategy which considers 3 different types of incoming virtual machines. The proposed algorithm focuses on the secure placement of VMs over physical machines. This work aims to minimise energy consumption.

In [38], the authors presented an open-source development model algorithm to address dynamic virtual machines’ placement in the cloud. ODMA(Open Source Development Model Algorithm) is one of the meta-heuristic approaches that is used in this work to consolidate several VMs into a reduced number of hosts. The objectives of this work are to minimise the number of active hosts, achieve load balancing and improve performance. In [39], the author has proposed a multi-agent-based resource optimisation algorithm is proposed which aims to solve the optimisation problem using parallel scheduling and a multi-agent system. The work proposes a mathematical model to find an optimal solution in parallel resources. Canosa-Reyes et al., [40] proposed energy tradeoff consolidation with contention-aware resource provisioning, here authors used containers to optimally place the jobs. Cloudsim was used for experimention purpose. The proposed method reduces resource contention and makes job placement more efficient with the energy-utilization tradeoff.

### Machine learning technquies

Ashawa et al. proposed the LSTM technique for load balancing to enhance cloud efficiency via resource allocation [41]. LSTM provided a dynamic resource allocation mechanism that evaluates the resource usage of an application using heuristics to determine the optimal additional resources to make available for that application. Based on the result, the proposed model shows the accuracy rate is enhanced by approximately 10–15%.

Ali Aghasi et al. employed the Q Learning technique to address virtual machine placement [42]. Reinforcement learning along with state action representation is utilised. The objectives of this work were to minimise energy consumption and reduce CPU temperature. In this generation of machine learning, various hybrid approaches have been proposed using machine learning and deep learning, like the Double Deep Q-network to improve network performance in cloud radio access networks [43]. This work [43] aims to improve performance by managing and optimising the load on network paths using Q-Network approaches. The result showcases an improvement in power consumption (Kwh) and network delay. Another work using deep learning was proposed in [44] to optimise energy efficiency and resource optimisation. This model tries to predict the best resource of a VM based on the prior performance in terms of CPU utilisation and power consumption. The work uses a deep reinforcement learning model for training and model prediction. The proposed model is compared with a greedy algorithm using power consumption and average waiting time as performance parameters.

Work has also been carried out in job scheduling. For example, Ibrahim Attiya et al. presented a hybrid job scheduling approach in cloud computing using a modified Harris Hawk Optimisation and simulated annealing algorithm [45]. This work aims to minimise the makespan and improve the convergence speed. Both standard and synthetic workloads were employed to analyze the performance of the this work.

Authors in [46] proposed a multi-objective task scheduling technique, based on Gaussian Cloud Whale Optimisation Algorithm (GCWOAS2) in cloud computing. A three-layer scheduling model was presented in this work. The goal is to reduce the operating cost of the system by minimising task completion time by effectively utilising virtual machine resources and maintaining the load balancing of each virtual machine. To develop the best scheduling scheme in the GCWOAS2 approach, an opposition-based learning mechanism is initially employed to establish the scheduling strategy. Then, to dynamically widen the search range, an adaptive mobility factor is provided. To improve the unpredictability of the search, a Whale Optimisation technique based on the Gaussian cloud model is presented.

To summarise, prior research shows that the meta-heuristic approaches listed above can identify an appropriate solution for VM scheduling in cloud computing. However, experiments were carried out using randomly generated data in some works and most of the work focused on two to three objectives without taking into account load balancing, SLA violation and execution time concurrently. The proposed work in this article focuses on multi-objective VM placement along with load balancing which was not addressed in the existing approaches.

### Motivation

The motivation of this work is to develop a new meta-heuristic algorithm to achieve better performance in the field of cloud computing. Where existing work as shown in the literature work uses traditional algorithms, this work proposes the Harris Hawk Optimisation (HHO) model to improve the performance of the cloud environment in terms of power consumption and utilisation of the system. The existing models are being compared with our proposed model to study the performance.

### Problem formulation

The cloud data centre in this work consists of N VM and K PMs. The resource requirements of VMs are CPU and RAM. The requirements of CPU and memory of VM_i_ are represented as $VM_{cpu_{i}}$. and $VM_{ram_{i}}$ respectively. The CPU and memory capacity of PMj are represented as $PM_{cpu_{j}}$ and $PM_{ram_{j}}$ respectively. Table 2 depicts the terminologies used in this work.

**Table 2:** Key terminologies.

Terminologies	Description
*VM* = {*vm*1,*vm*2,…*vmn*}	Set of VMs
*PM* = {*pm*1,*pm*2,…*pmk*}	Set of PMs
*VM* = (*pm*)	Set of VMs hosted by a PM *j* ∈ *PM*
*r*_*pm*_(*t*) = *L*_*pm*_(*t*)	VM resource needs to be aggregated at a PM
*r*_*vp*_(*t*) = *L*_*vp*_(*t*)	The VM resource demands placed on PM
*Capacity*_*pm*_	PM capacity (e.g., CPU power, memory)

Each PM has enough capacity in this cloud data centre to allocate a set of VMs. Let *r* = (*r*_*pm*_,*pm* ∈ *PM*) denote the VM placement approach satisfying the resource allocation policy is feasible i.e. resources allocated to every VM are fewer than the overall capacity of the PM as represented in Eq 1.


$$\sum_{pm:pm\in PM}W_{pm}\cdot r_{pm}\leq 0,$$ (1)

Where *W*_*pm*_ represents the se’ver’s willingness to offer resources or performance weight. Considering the proposed VMP method, let *γ*_*pm*_ be the fairness among the association of PMs. Once *γ*_*pm*_ = 1, the utility function of the pm is represented as *UT*_*pm*_(*r*_*pm*_(*t*)) = *W*_*pm*_*log r*_*pm*_(*t*). Next, maximising the cumulative utilities of all PMs in the data centre is expressed as:

$$\max\sum_{pm\in PM}W_{pm}\;log\;r_{pm}(t)$$ (2)

### Virtual machine placement problem statement

Let *L*_*vp*_(*t*) symbolise the load of *VM i* which is hosted on physical machine pm and *L*_*pm*_(*t*) denote the aggregate load of PM, the following condition (3) must be satisfied:

$$L_{pm}(t)=\sum_{i:i\in VM(PM)}L_{vp}(t)$$ (3)

Here *L*_*vp*_(*t*) is the VM’s load requirements as the d dimensional vector, where d = 2 when CPU and memory are considered, *L*(*t*) is given by

$$L(t)=\left(VM_{cpu_{i}},VM_{ram_{i}}\right)$$ (4)

Further, *Capacity*_*pm*_ is defined as the available server capacity on PM *j* ∈ *PM* regarding its CPU and RAM. The following formula must hold true to confirm that the overall load on any PM is not more than its capacity.


$$\sum_{vm:vm\in VM(PM)}L_{vp}(t)\leq \;Capacity{\;}_{pm}$$ (5)

Typically, optimal placement of virtual machines on servers and turning off other servers leads to maximisation of utilisation and minimising server power consumption. To reflect this, in our analysis, the following equation is utilized:

$$\left(Y_{1}\right):\max\sum_{pm:pm\in PM}UT_{pm}\left(L_{pm}(t)\right)$$ (6)

Subject to

$$\sum_{vm:vm\in VM(pm)}L_{vp}(t)=L_{pm}(t),\forall \;pm\in PM,$$ (7)

$$\sum_{vm:vm\in VM(pm)}L_{vp}(t)\leq \;Capacity{\;}_{pm},\forall \;pm\in PM$$ (8)

$$\;Over\;L_{vp}(t)\geq 0,vm\in VM,pm\in PM$$

Based on the constraints below, a single PM can host a set of VMs:

$$\sum_{i}^{n}VM_{cpu_{i}}\leq PM_{cpu_{j}},\forall \;vm_{i}\in VM,\;and\;pm_{j}\in PM$$ (9)

$$\sum_{i}^{n}\;VM_{ram_{i}}\leq PM_{ram_{j}},\forall \;vm_{i}\in VM,\;and\;pm_{j}\in PM$$ (10)

The above equation ensures that the total resources used by a group of VMs should not surpass the CPU and memory capacities of PM.

When only CPU and RAM are considered, the PM resource utilisation problem (*Y*_1_) will be equivalent to:

$$\left(Y_{1}^{\prime }\right):\max\sum_{pm:pm\in PM}UT_{pm}\left(PM_{cpu_{j}}(t)\times PM_{ram{\;}_{j}}(t)\right)$$ (11)

Subject to

$$\sum_{vm:vm\in VM(PM)}PM_{cpu_{j}}(t)\leq \;Capacity{\;}_{pm}^{cpu},\forall \;pm\in PM$$ (12)

$$\sum_{vm:vm\in VM(PM)}PM_{ram{\;}_{j}}(t)\leq \;Capacity{\;}_{pm}^{ram},\forall \;pm\in PM$$ (13)

$$\;Over\;L_{vp}(t)\geq 0,vm\in VM,pm\in PM$$

To facilitate the subsequent derivation of the formula, let *r*_*pm*_(*t*) = *L*_*pm*_(*t*) To maximise the data ce’tre’s’ overall aggregate utilities and find the best solution, a Lagrange function is defined as:

$$\begin{array}{c} \begin{array}{l} LR\left(r_{vp},r_{pm},\gamma ,\beta \right)\\ =\sum_{pm:pm\in PM}\left\{UT_{pm}\left(r_{pm}(t)\right)\;+\;\gamma_{pm}\left(\sum_{vm:vm\in VM}r_{vp}(t)\;-\;r_{pm}(t)\right)\right\} \end{array}\\ +\;\;\;\sum_{pm:pm\in PM}\beta_{pm}\left(\;Capacity{\;}_{pm}\;-\sum_{vm:vm\in VM}r_{vp}(t)\;-\;\varepsilon_{pm}^{2}\right) \end{array}$$ (14)

Where *γ* = (*γ*_*pm*_, *pm* ∈ *PM*) and *β* = (*β*_*pm*_, *pm* ∈ *PM*) are Lagrange multiplier vectors,

$\varepsilon^{2}=\left(\varepsilon_{pm}^{2},pm\in PM\right)$ is the relaxation factor vector. Let *γ*_*vm*_ denote the load requirement of the virtual machine vm. Let *β*_*pm*_ be the available capacity of the physical machine pm. Let the resource occupied by all VMs on physical machine pm be expressed as $\sum_{vm:vm\in VM(pm)}r_{vp}(t)$ and $\sum_{vm:vm\in VM(pm)}r_{vp}(t)\;\cdot \;\varepsilon_{pm}^{2}\geq 0$ represents the enduring resources present on the physical machine pm.

According to Eq (7), we obtain:

$$\begin{array}{c} \begin{array}{l} LR\left(r_{vp},r_{pm},\gamma ,\beta \right)\;\\ =\;\sum_{pm:pm\in PM}\left\{UT_{pm}\left(r_{pm}(t)\right)-\gamma_{pm}r_{pm}(t)\right\} \end{array}\\ +\;\sum_{pm:pm\in PM}\sum_{vm:vm\in VM(PM)}r_{vp}(t)\left(\gamma_{pm}-\beta_{pm}\right)\\ +\;\sum_{pm:pm\in PM}\beta_{pm}\left(\;Capacity{\;}_{pm}-\varepsilon_{pm}^{2}\right) \end{array}$$ (15)

Where $\;Capacity{\;}_{pm}-\varepsilon_{pm}^{2}$ represents the occupied resource of physical machine pm. To increase the PM utilisation and minimise server energy consumption, optimal packing is performed to place virtual machines then $\beta_{pm}\left(\;Capacity{\;}_{pm}-\varepsilon_{pm}^{2}\right)$ can be considered as the gains of PM from packing.

To save energy any PMs that are not’ in use should be turned off. The power consumption of active PMs is formulated as follows:

$$\sum_{j=1}^{k}PC\left(PM_{cpu_{j}}\right)\;=\;\left\{\begin{array}{c} PM_{idle\;}\;+\;\left(PM_{max}-PM_{idle\;}\right)\;\times \;PM_{cpu_{j}},\;\text{if}\;PM_{cpu_{j}}\;>\;0\\ 0,\;otherwise\; \end{array}\right.$$ (16)

where P_idle_ is the idle-state power of PM_j_ , P_Max_ is the maximum power of PM_j_ , and P_cpuj_ is the percentage value ∈ [0, 1] that denotes the CPU utilisation.

### Harris Hawk Optimisation for virtual machine placement

Heidari and Mirjalili et al. developed the Harris Hawks Optimisation Technique (HHO), a novel optimisation algorithm [47]. The algorithm mimics the natural behaviour and hunting strategy of Harris Hawks known as surprise pounce. The hawks collaborate to attack from many directions to startle the victim. Harris Hawks reveal a variety of pursuit methods dependent on the nature of the schemes and the victim’s’ evasive patterns. Exploration and exploitation tactics are proposed by the conventional HHO algorithm which is driven by exploring prey, surprise pounce, and Harris Haw’s’ particular attacking approach. The Harris Hawks are the candidate solutions and the targeted prey in each phase is the best candidate solution (nearly the optimal one). The exploration phase, transition from exploration to exploitation phase, and exploitation phase are the three phases of the HHO algorithm and are described below.

#### i) Exploration phase

**Fig 2:**
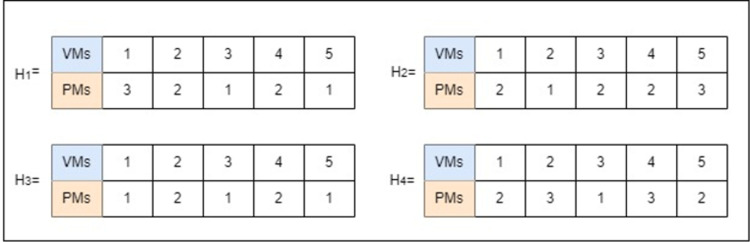
Example of four feasible applicable solutions.

All Harris Hawks are considered solutions during this phase denoted as a solution matrix H; is a 2 × *N* matrix, where N is the number of VMs. Fig 2 depicts 4 feasible solutions where four hawk agents’ solutions assign 5 VMs to 3 PMs. The four solutions are represented by the set *H = H*_*1*_, *H*_*2*_, *H*_*3*_, *and H*_*4*_. According to the number of VMs, all solutions are reviewed and ranked in ascending order of PMs that are utilised in this solution [12]. The order of sorting is H_3_, H_1_, H_4_, and H_2_. The best solution is H3 because it has the minimum number of PMs, which consumes less power.

The solution matrix of a single hawk H is defined in Eq 17. If there are *N* VMs to be allocated to *J* PMs, the value of the component of H of the matrix represents the PM index to host the VM number *n*.


$$H=\left[\begin{array}{c} y_{1}^{i},y_{2}^{i},y_{3,}^{i},\ldots .y_{N}^{i}\\ y_{1,1}^{i,j},y_{1,2}^{i,j},y_{2,3}^{i,j},\ldots .y_{1,N}^{i,j} \end{array}\right]$$ (17)

The corresponding variable $y_{1,n}^{i,j}$ is the value *j*, ∀ *j* ∈ [1, *J*], if the virtual machine *VM*_*i*_ is assigned to *PM*_*j*_ ∈ *PM*. Variable $y_{n}^{i}$ denotes the virtual machine *VM*_*i*_ ∈ *VM*.

The fitness value is determined for all these feasible solutions created on the desired prey in each iteration. To replicate Harris Hawk’s’ exploring abilities in the search space chosen and updating the solution matrix is according to two techniques as specified in Eq 18.


$$H(t+1)\;=\;\left\{\begin{array}{cc} H_{rand\;}(t)-r_{1}|H_{rand\;}(t)-2r_{2}H(t)| & p\geq 0.5\\ \left(H_{\rho }(t)-H_{s}(t)-r_{3}\left(LB+r_{4}(UB-LB)\right)\right. & p<0.5 \end{array}\right.$$ (18)

Where *H*(*t* + 1) is the hawk’s candidate solution/position in the second iteration t, *H_ρ_*(*t*) represents the best solution matrix/prey position and *H*_*rand*_(*t*) is the random solution selected in the present population.*H*(*t*) represents the position vector of hawks in the present iteration t. r_1_, r_2_, r_3_, r_4_ and p are the random scaled factor within the range [0,1]. UB and LB are the upper bound and lower bounds of the variables, *H*_*s*_(*t*) denotes the average number of solutions. The index of the first row is updated according to the hawk agent solutions of the best and random solution as per Eq 18.

Hawk placements are generated because of this method inside UB and LB depending on 2 rules 1) Build solutions using a randomly chosen hawk from the present population as well as other hawks. 2) Construct solutions depending on the location of the prey, the average hawk position, and random scaled variables. While r3 is a scaling factor, if the value of r4 approaches 1 it will aid in increasing the rule’s’ unpredictability. An arbitrarily scaled measure length is added to LB in this rule. More diversification strategies to investigate other sections of the feature space are examined using a random scaled component. The average hawk position (solutions) is formulated as follows:

$$H_{s}(t)\;=\;\frac{1}{M}\sum_{i=1}^{M}H_{i}(t)$$ (19)

Where, *H*_*s*_(*t*) is the current iteration’s’ average number of solutions. M denotes all possible solutions. *H*_*i*_(*t*) indicates the location of every solution in iteration t.

The updated indexes in the hawk solutions should be in the range of [1,J]. If the updated index is outside of the range then the algorithm recalculates it as follows:

$$y_{1,n}^{i,j}(t+1)\;=\;\left\{\begin{array}{cc} y_{1,n}^{i,j}(t+1)mod\;J, & \;if\;y_{1,n}^{i,j}(t+1)\notin [1,J]\\ y_{1,n}^{i,j}(t+1), & \;otherwise \end{array}\right.$$ (20)

#### ii) The transition from exploration to exploitation

Based on the energy of the prey, this phase shows how HHO moves from exploration to exploitation. HHO posits that the energy of the prey is gradually depleted as a result of the fleeing activities. R_0_ is the initial energy range between [–1,1] as expressed in Eq 21.


$$R=2R_{0}\left(1-\frac{t}{T}\right),R_{0}\in [-1,1]$$ (21)

Where t is the current iteration and T represents the maximum number of iterations.

#### iii) Exploitation phase

The exploitation phase is marked completed by utilising 4 methods/ways at parameter sets. These methods are created on the position that was discovered during the exploration phase. The prey, on the other hand, tries to flee often despite the hawk’s ‘efforts to track it down and trap it. HHO exploitation uses four different techniques to imitate the hawks’ attacking style. Hard besiege, Soft besiege, soft besiege with progressive rapid dives, and hard besiege with progressive rapid dives are the four methods. These methods depend on 2 factors r and |R|. Where R represents the prey” fleeing energy and r is the likelihood of escaping with *r* < 0.5 indicating a better chance of the prey escaping successfully and *r* ≥ 0.5 indicating an unsuccessful escape. The following is a summary of these approaches:

In the soft besiege approach, where *r* ≥ 0.5 *and* |*R*| ≥ 0.5, while the hawks gently round on the victim causing it to lose extra energy before completing the surprise pounce the prey still has some energy to flee. Soft besiege is mathematically formulated in Eq 22.


$$H(t\;+\;1)\;=\;\Delta H(t)\;-\;R|JH_{\rho }(t)\;-\;\text{H}(t)\;|$$ (22)

$$\Delta H(t)\;=\;H_{\rho }(t)\;-\;\text{H}(t)$$

$$J=2\left(1\;-\;r_{5}\right),\;r_{5}\in [0,1]$$

Where Δ*H*(*t*) is the difference between the prey” position vector and the current location in iteration t, J is the ‘ prey” jump strength, and r_5_ is a random variable.

In the hard besiege, where *r* ≥ 0.5 *and* |*R*| < 0.5, the prey is exhausted and has a slight chance of escaping. In this situation, the hawk barely encompasses the target before launching the ultimate surprise pounce. Accordingly, the solution is updated using Eq 23.


$$H(t+1)\;=\;H_{\rho }(t)\;-\;R|\Delta H(t)|$$ (23)

In soft besiege with progressive rapid dives method with *r* < 0.5 *and* |*R*| ≥ 0.5, the prey has enough energy to flee. The hawk manoeuvres deftly around the victim and descends tolerantly before the attack. This is referred to as a clever soft besiege, in which the hawk’s ‘location is updated in two phases. In t^he^ 1^st^ stage, the hawks approach the prey by calculating the prey” next move as in Eq 24.


$$K\;=\;H_{\rho }(t)\;-\;R\;|\;C\;H_{\rho }(t)\;-\;H(t)|$$ (24)

C represents the jump power of prey. The hawk then determines whether to dive in the second stage depending on a comparison of the prior dive and the likely outcome. If it is not,’ the hawks will produce an uneven dive based on the Levy Flight (LF) notion as expressed in (25)

L=K+Q*LF(d) (25)

Where d is the dimension of solutions, Q is the random vector of size 1*d. LF is the Levy Flight function designed using Eq 26

$$LF(d)\;=\;0.01\;*\;\frac{y\;*\;\sigma }{|z{|}^{\frac{1}{\beta }}},\;\sigma \;=\;{\left(\frac{\tau (1\;+\;\beta )\;*\;\sin\left(\frac{\pi \beta }{2}\right)}{\tau \left(\frac{1\;+\;\beta }{2}\right)\;*\;\beta \;*\;2^{\left(\frac{\beta -1}{2}\right)}}\right)}^{1/\beta }$$ (26)

Where *β* is the default constant and y, z are the random variables between [0, 1]. As a result, a method for updating the Harris Hawk’s ‘locations with advanced quick dives may be devised as

$$H(t\;+\;1)\;=\;\left\{\begin{array}{l} K\;if\;F(K)\;<\;F(H(t)\\ L\;if\;F(Z)\;<\;F(H(t) \end{array}\right.$$ (27)

Here, K and L are performed using Eqs 24 and 25.

In the last approach, hard besiege with progressive rapid dives where *r* < 0.5 *and* |*R*| < 0.5, the prey has no energy to flee, therefore the Harris Hawks try to approach the prey by diving quickly before making a surprise pounce to grab it. The hawk movement’ in the situation is stated in Eq 27

Where K and L are as follows

$$K\;=\;H_{\rho }(t)\;-\;R\;\left|\;CH_{\rho }(t)\;-\;H_{s}(t)\;\right|$$ (28)

L=K+Q*LF(d) (29)

The parameters used in this work are presented in Table 3.

**Table 3:** Parameters of HHO.

Name of the parameter	Adopted Value
Number of search agents	50
Dimension	Number of VMs
Lower bound	1
Upper bound	800
Maximum Iterations	100

#### Objective function

After the hawk agents’ solutions have been updated the solutions are evaluated to choose the best one *H*_*ρ*_. Only one HW (Hawk) solution is chosen as the best where HW denotes the number of hawk agents. The algorithm compares the solutions based on criteria *e*_*1*_ *in* Eq 30, this shows the amount of energy consumed by this solution. The best solution is the *H*_*ρ*_ solution with the least power consumption and the fewest number of PMs used [12]. The objective function is formulated as:

$$min.\;e_{1}(H)\;=\;\sum_{j=1}^{J}PC\left(P_{CPU_{j}}\right)$$ (30)

Pseudocode 1: Harris Hawk Optimisation

**Input**: Set of virtual machines N and physical machines J

**Output**: 2 × *N* allotment matrix mapping N virtual machines to J physical machines as *H*_*ρ*_

1. **Initialisation**: *HW*: = 50, *ItrE*: = 100, *it*: = 0, *LB*: = 1, *UB*: = 800
2. **while *it<ItrE* do**
3.  Produce *HW* × 2 × *N* hawk solution matrices
4.  Assess the solutions and assign the best solution using the *best solution*: *= H*_*ρ*_
5.  initialise *a*: *=* 1 for each iteration of the search
6.  **if** the solution ≠ *H*_*ρ*_ **then**
7.   Update the present solution H*a* using Eq (18)
8.  **if** the number of hawks *a* < HW **then**
9.   set *a*: *= a+1* and go to step 6
10.  Assess the fitness of the HW solutions using Eq 30 and assign the best solution to *H*_*ρ*_
11. **Return** 2 × *N* allotment matrix *H*_*ρ*_ the best solution.

**Fig 3:**
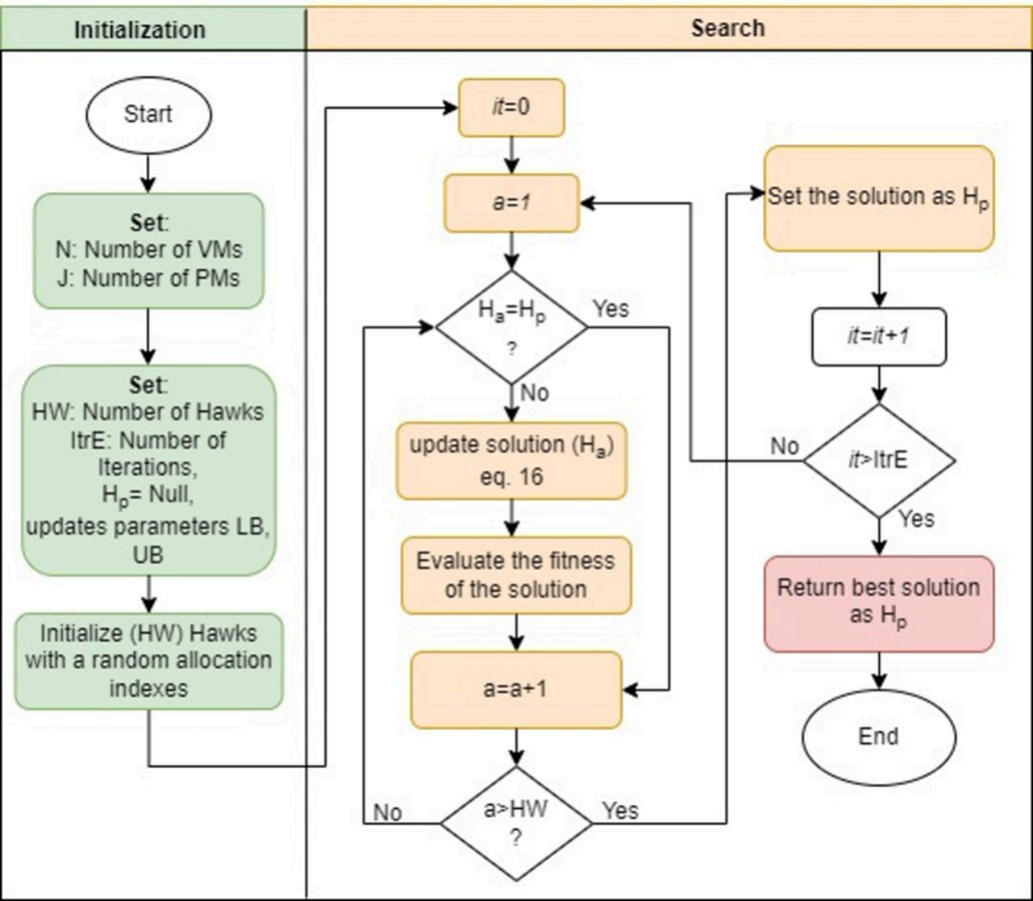
Workflow process of HHO algorithm.

The pseudocode of HHO is depicted in Pseudocode 1 and the workflow process of the HHO is represented in Fig 3. The hawk agents start with randomly distributed indexes and then analyze their solution to determine the best *H*_*ρ*_ . The hawk agents then update their solutions based on the best option that has been chosen thus far. The optimal solution is then presented as matrix indices which map the VMs to the minimum number of PMs at the end of each iteration. The HHO algorithm has the advantages of simple operation, fewer adjustment parameters, ease of implementation and use of communication between hawks to improve the global search capability. But for higher dimensional problems it may have low converge performance.

### Load balancing

To perform the load balancing in the data centre, host overload and host underload detection mechanisms are incorporated.

#### Host overload detection

Each host periodically executes an overload detection algorithm to de-consolidate VMs when needed to avoid performance degradation and SLA violation. In this work, we used the IQR (Interquartile Range) to detect the overloaded machines in the data centre and the Maximum Correlation (MC) policy [48] is applied to choose the VMs to be migrated from overloaded hosts to some other host. MC selects the VMs having a maximum correlation of the CPU consumption with other virtual machines.

IQR is a method for setting an adaptive CPU utilisation threshold based on robust statistics. In descriptive statistics, the IQR, also called the midspread or middle fifty, is a measure of statistical dispersion. It is equal to the difference between the third and first quartiles: IQR = Q3—Q1. Unlike the total range, the interquartile range is a robust statistic having a breakdown point of 25% and thus is often preferred to the total range. Using IQR the CPU utilisation threshold is defined in Eq 31.

$$T_{u}\;=\;1\;-\;s\;*\;IQR$$ (31)

where s is a parameter of the method defining the safety of the method.

#### Host underload detection

First, all overloaded hosts are identified using the overload detection technique and the VMs that will be migrated are assigned to the destination hosts. The system then attempts to deploy all the VMs from this host onto other hosts with minimal utilisation relative to the other hosts while ensuring that they are not overloaded. The VMs are configured for migration to the determined destination hosts if such a placement is possible. To save energy the source host is put to sleep mode once the migrations are done. The source host is maintained operational if all the VMs from the source host cannot be moved to other hosts. For all non-overloaded hosts, this step is done repeatedly.

### Complexity analysis of the proposed method

In further discussion V and P denote the number of VMs and PMs respectively. Each iteration consists of two steps. The first step is to update the solution. The first step includes applying Eq 19 to each column of all solution matrices. As a result of modifying the VM’s resource utilisation the PM’s CPU and RAM utilisation may rise or decrease. Thus, the time complexity of this step is O(V).

Second, to balance the load across the data centre, overloaded PMs are collected as discussed in the load balancing section. In the worst case when the PM is overloaded, selected VMs are migrated to other PMs. If the underloaded PM is found the PM is switched to sleep mode by migrating the VMs to some other PM. The time complexity of this task is *O*(*V* × *P*). The PM’s CPU and RAM usage increases and decreases with each VM migration from one PM to another according to Eq 30. Thus, the fitness function can be computed by summing up the power consumption of each PM in the time complexity of O(P).

So, the worst-case total time complexity of the 2 steps and the fitness computation of each iteration is *O*(*V* + (*V* × *P*) + *P*) . As algorithm 1 has ItrE iterations, the worst time complexity is *O*(*HW* × ItrE × V × P) for HW hawks.

## Experiment and comparisons

This section covers the experimental setup, performance measurements, and experimental outcomes.

### Experimental setup

To test the proposed method we used the CloudSim 3.0 toolkit simulator. Cloudsim offers a variety of virtual machine provisioning methodologies and Virtualised resources. We used real workload traces from PlanetLab to conduct the experiment. PlanetLab is a component of the CoMon project, which collects CPU utilisation from over 1000 virtual computers running on various hosts in over 500 locations across the world. We employed four distinct types of virtual computers in our test setup: Micro, Small, Medium, and Extra-Large instances. A total of 600 HP ProLiant G4 and HP ProLiant G5 heterogeneous hosts have been deployed. Table 4 lists the characteristics of these servers.

**Table 4:** Characteristics of servers.

Host Type	Depiction
HP ProLiant G4	1860 MIPS, 2 GB network bandwidth, 4GB RAM and 1.5 GB storage
HP ProLiant G5	2660 MIPS, 4 GB network bandwidth, 4GB RAM, and 2.5 GB storage

### Performance natrix and results

To assess the proposed approach against other algorithms the workloads depicted in Table 5 were used. It shows the workload number and the number of VMs in each of the workloads. Each of the workload files contains CPU utilisation values measured every 5 minutes in PlanetLab’s VMs [49]. These trace files contain traces of CoDeeN, the Coral Content Distribution Network, and Open DHT. The experiment was carried out using the workloads specified and the average result of these workloads was used to evaluate different algorithms based on the five metrics described below.

**Table 5:** Traces of workload from PlanetLab.

Workload		No. of VMs
20110306	W1	898
20110309	W2	1061
20110325	W3	1078
20110412	W4	1054
20110420	W5	1033

Experiments were carried out in two scenarios. Firstly, simulation is carried out using the workloads specified and the average result of these workloads is used to evaluate different algorithms based on the metrics under various workloads and task utilisation. For the second scenario, the performance of the proposed algorithm is done with scaling resources to study the performance in underloaded and overloaded conditions.

#### a) Energy consumption

**Fig 4:**
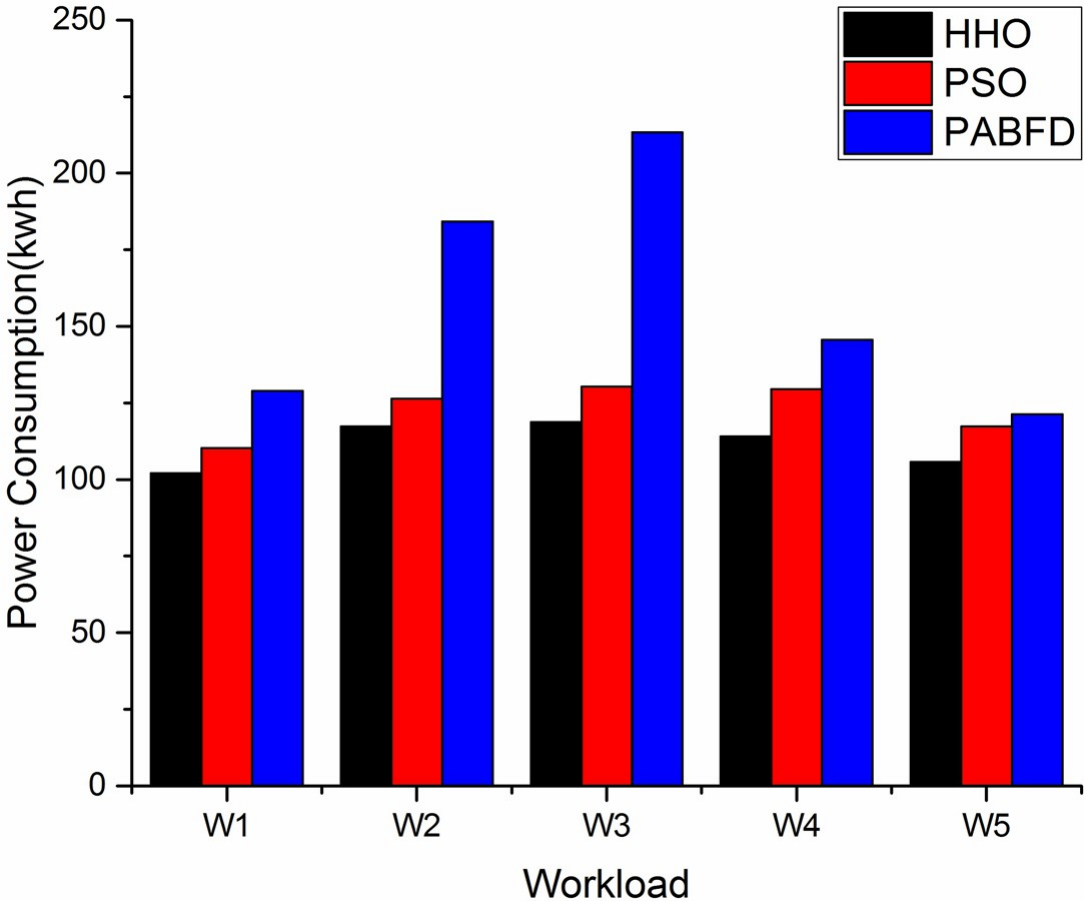
Energy consumption of data centre using different techniques using Scenario 1.

**Fig 5:**
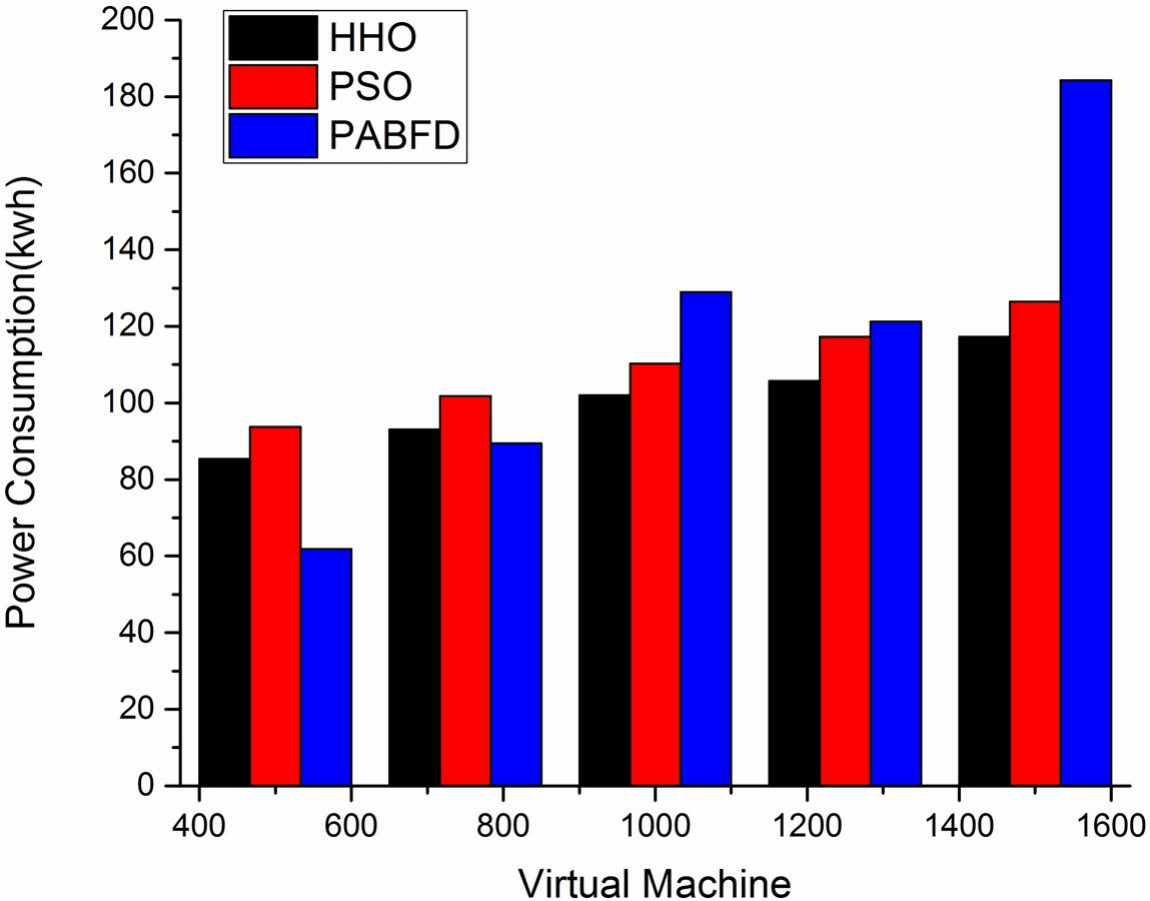
Energy consumption of data centre using different algorithms using Scenario 2.

Energy Consumption represents the total amount of energy/power consumed by all the data centre’s PMs. Fig 4 depicts the energy consumption of the algorithms using scenario 1. As the instance size grows, the power consumption gradually increases. The result shows that HHO decreases average power consumption by 9% and 27% compared to PSO (Particle Swarm Optimisation) and PABFD (Best Fit Decreasing) respectively. Fig 5 shows the study with scaling resources using scenario 2 where the proposed algorithm shows the least energy consumption. The simulation is supported by two machines HP ProLiant ML110 G4 (Intel Xeon 3,040, 2 cores, 1,860 MHz, 4 GB) and HP ProLiant ML110 G5 (Intel Xeon 3,075, (2 cores, 2,660 MHz, 4 GB) [49] as defined in Cloudsim3.0. Table 6 shows the power consumption by a machine under different utilisation levels.

**Table 6:** Energy and utilisation of machine power model.

Server Utilisation	0%	10%	20%	30%	40%	50%	60%	70%	80%	90%	100%
**HP ProLiant G4**	86	89.4	92.6	96	99.5	102	106	108	112	114	117
**HP ProLiant G5**	93.7	97	101	105	110	116	121	125	129	133	135

#### b) Resource utilisation

**Fig 6:**
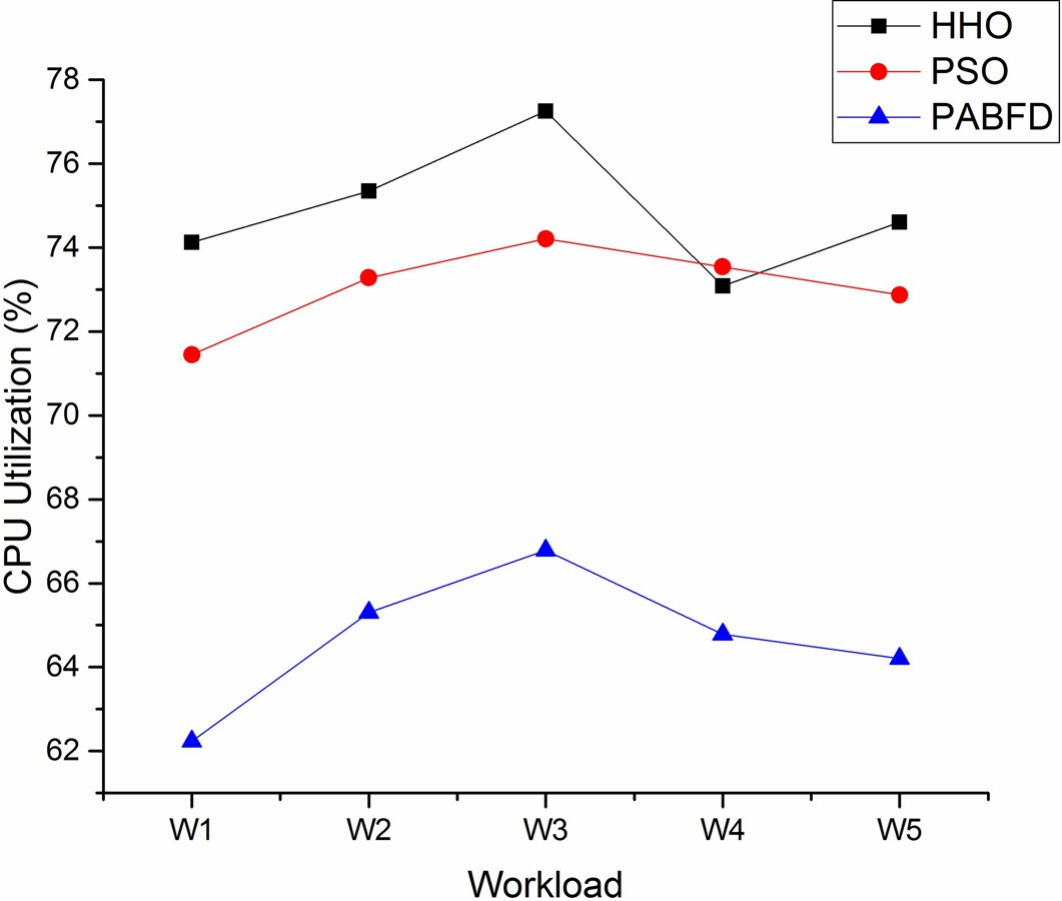
CPU utilisation in the heterogeneous environment using Scenario 1.

**Fig 7:**
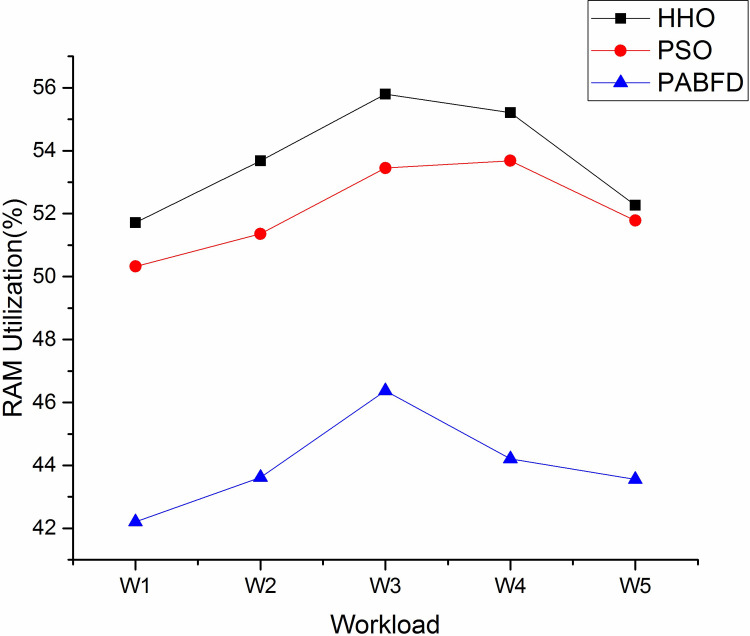
RAM utilisation in the heterogeneous environment using Scenario 1.

**Fig 8:**
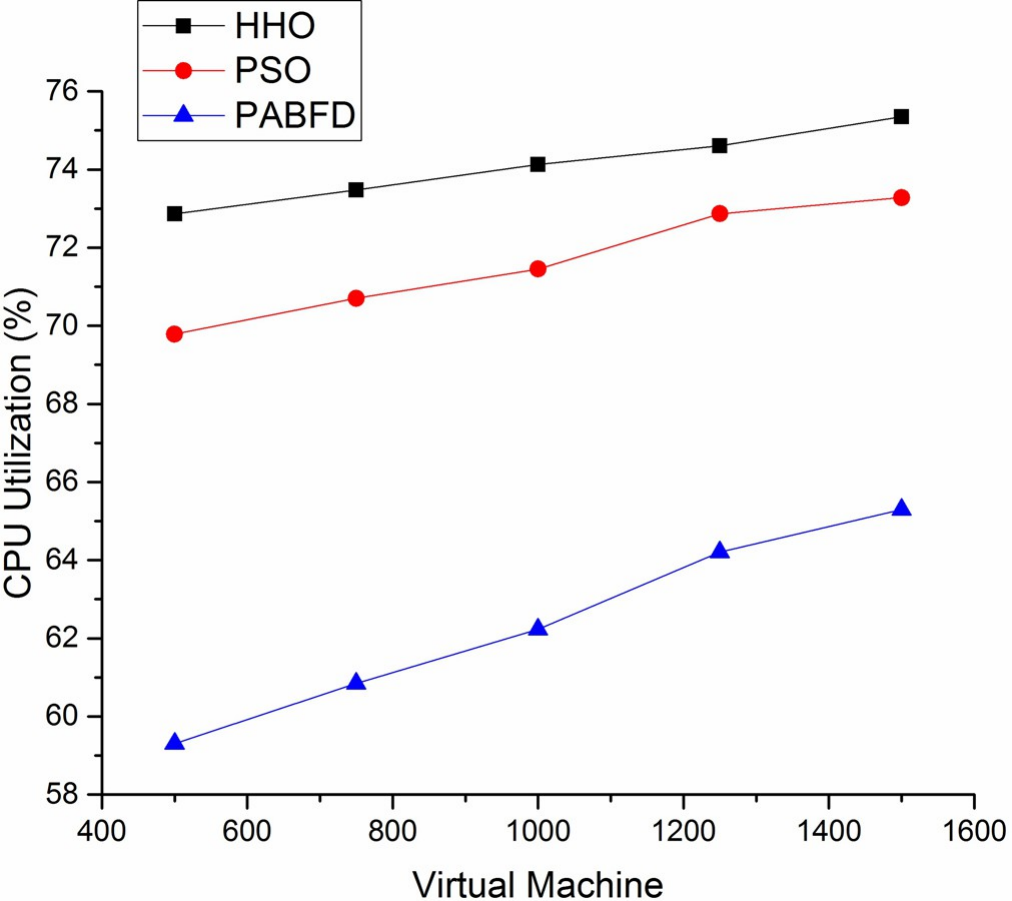
CPU utilisation using Scenario 2.

**Fig 9:**
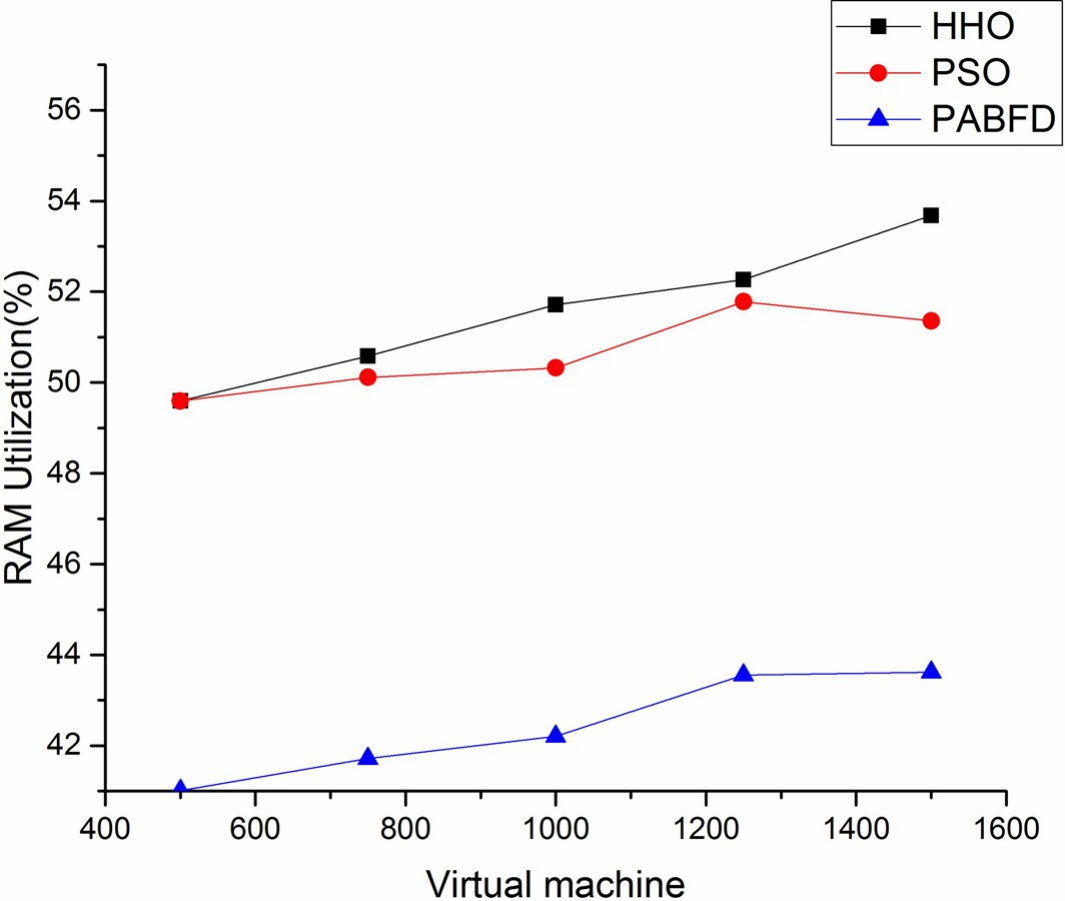
RAM utilisation using Scenario 2.

Figs 6 and 7 shows the resource utilisation of CPU and memory for the PMs to host VMs using scenario 1. It is observed from the result that the average CPU utilisation of HHO is higher by 3% and 12% compared to PSO and PABFD respectively. Similarly, the memory utilisation of HHO is 4% and 17% higher than PSO and PABFD. Figs 8 and 9 shows the performance study of CPU and RAM utilisation with increasing virtual machines using scenario 2, where the proposed algorithm improves the CPU and RAM utilisation as compared to existing models.

#### c) Number of VM migrations

**Fig 10:**
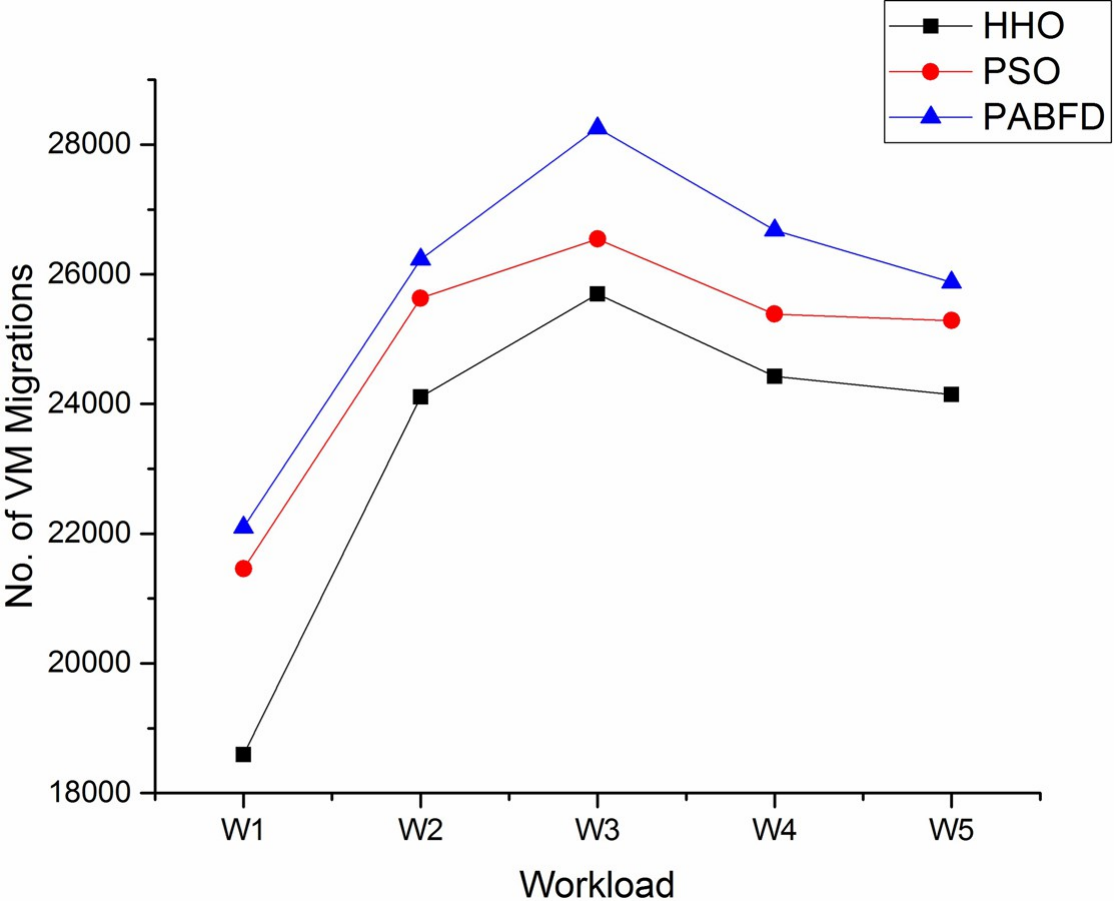
Number of VM migrations in the data centre using scenario 1.

**Fig 11:**
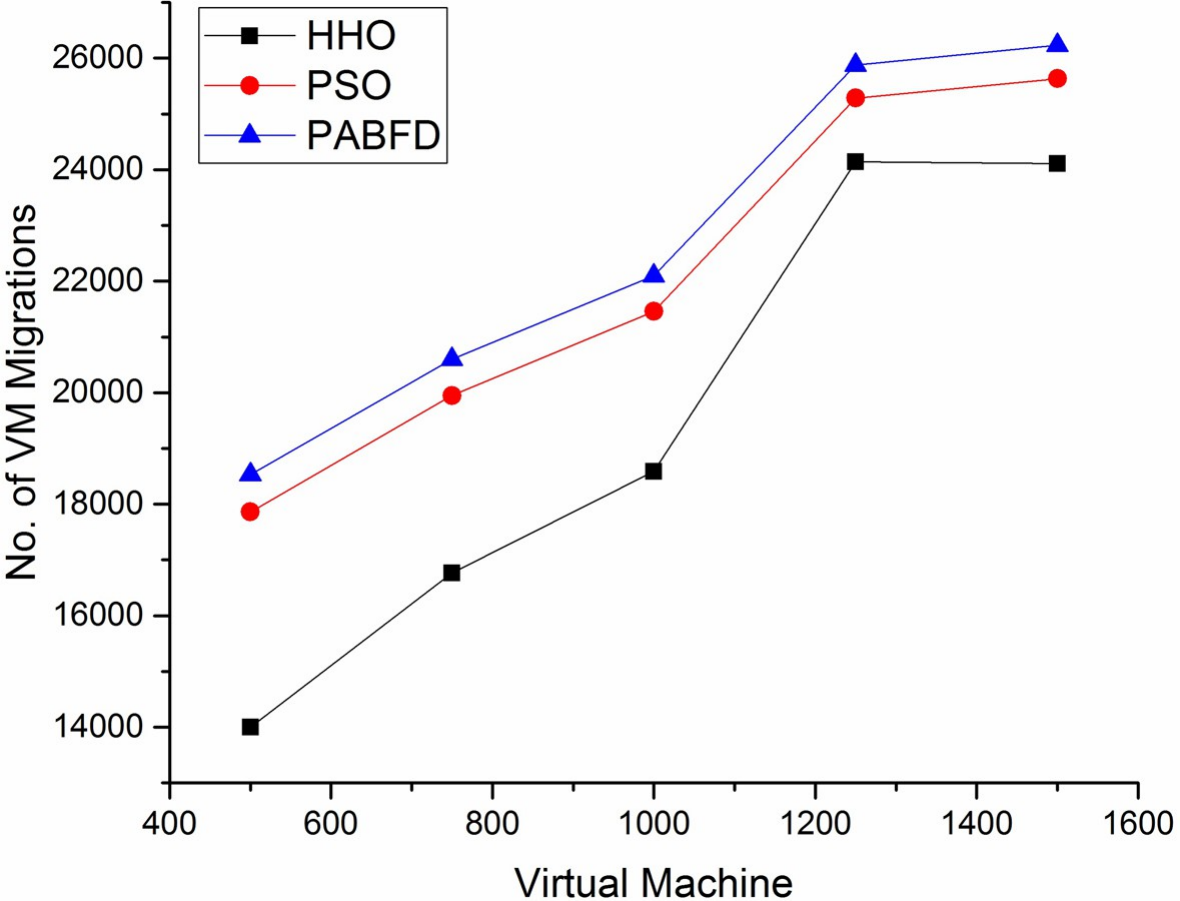
Number of VM migrations in the data centre using scenario 2.

The number of VM migrations is another evaluation metric. As the number of requests increases, some of the PMs get overloaded, to balance the load across PMs some of the virtual machines are selected and migrated to other physical machines of the data centre as discussed in the load balancing section. According to Fig 10, the number of VM migrations in HHO is less compared to other algorithms using scenario 1, since HHO places the VMs on an apt and lesser number of PMs which reduces the chance of migrating VMs between PMs. Fig 11 shows a decrease in the number of VM migrations as compared to existing algorithms using scenario 2.

#### d) SLA violation

**Fig 12:**
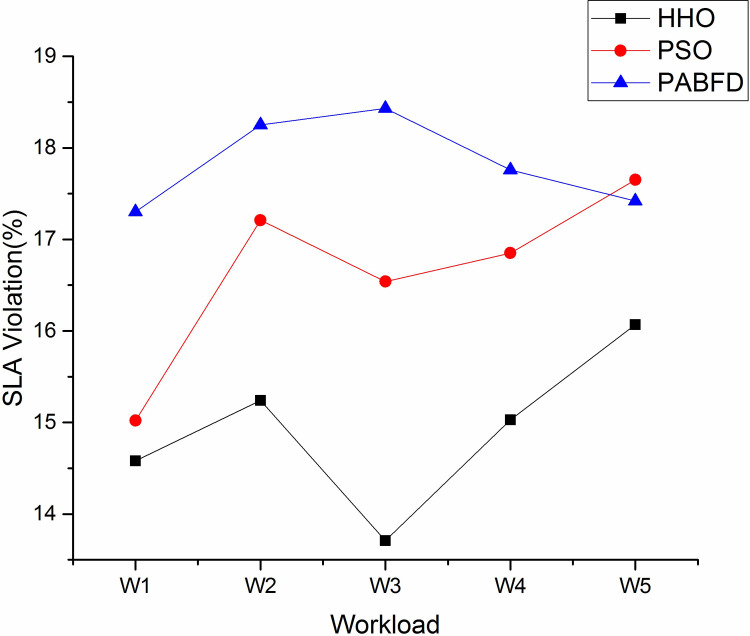
Average SLA violation (%) through different algorithms using scenario 1.

**Fig 13:**
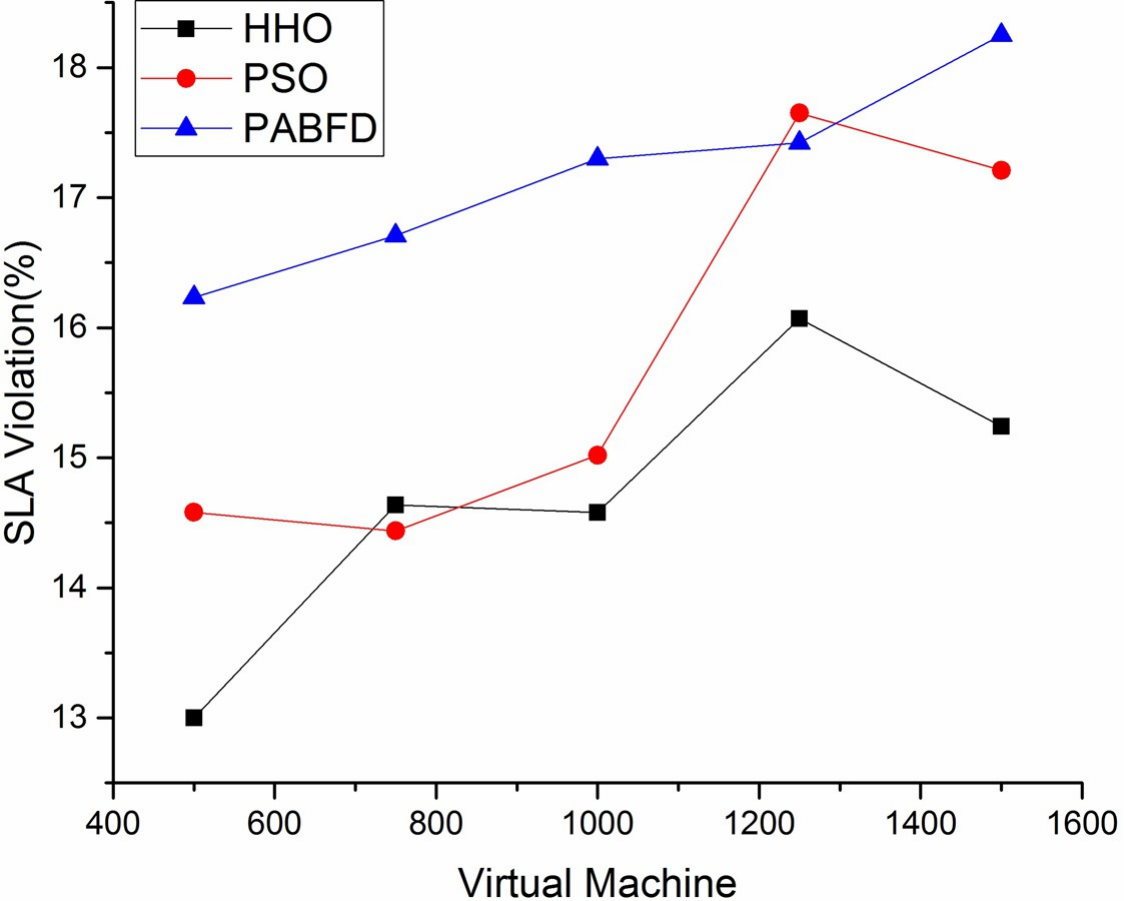
Average SLA violation (%) through different algorithms using scenario 2.

The average SLA violation of different algorithms using scenario 1 is depicted in Fig 12. As shown. the average SLA violation of the HHO algorithm is less by 8% and 16% compared to PSO and PABFD respectively. Fig 13 shows an improvement in SLA violation with increasing resources as compared to existing algorithms using scenario 2.

#### e) Execution time- VM reallocation

**Fig 14:**
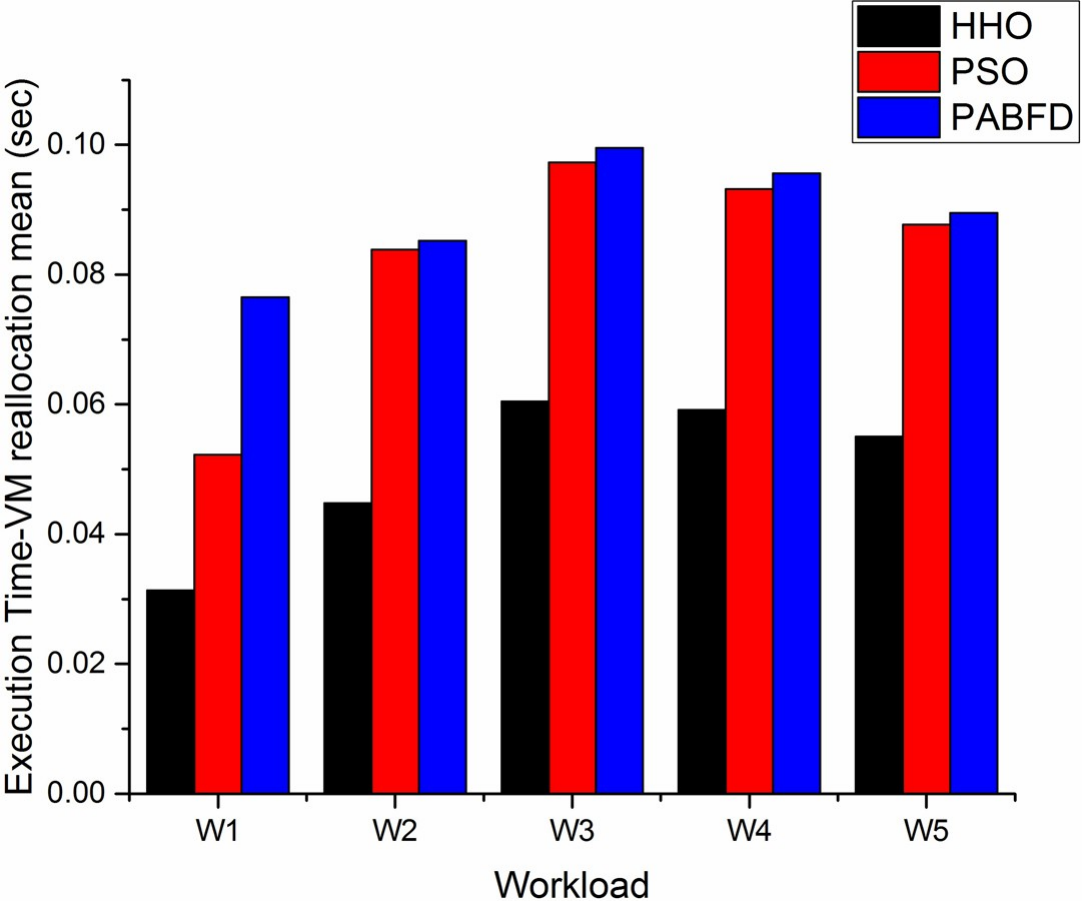
VM reallocation mean (sec) using scenario 1.

**Fig 15:**
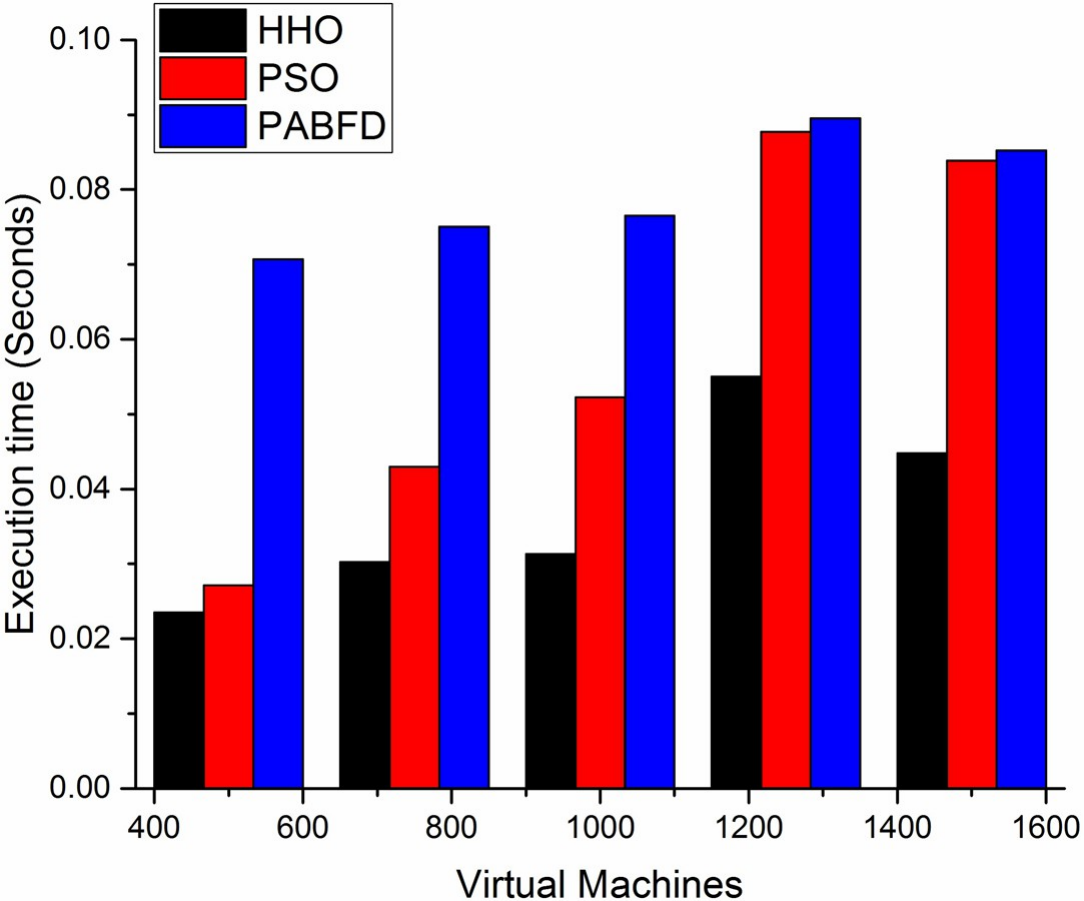
VM reallocation mean (sec) using scenario 2.

Fig 14 shows the mean execution time required for VM reallocation using scenario 1. As shown in the result, the mean reallocation time of HHO is less compared to PSO and PABFD. Since the number of VM migrations is less in HHO, as a result, the mean execution time for VM reallocation is also less. It is observed that HHO execution time is reduced by 3.2% and 4% compared to PSO and PABFD respectively. Fig 15 shows the lower mean reallocation time of HHO using scenario 2.

## Conclusion

Developing a multi-objective virtual machine placement strategy has been a prominent research subject as virtualisation technology has advanced. This work brings in the following significant contributions: 1) multi-objective model formulation of dynamic VM placement and 2) a Harris Hawk-based meta-heuristic algorithm to optimise virtual machine placement according to energy and resource constraints. HHO has been previously used by researchers for improving performance in various domains like parallel scheduling [49] and memory management [50].

The proposed algorithm was evaluated through a set of tests. A total of 600 heterogeneous hosts were deployed and real-time workload from PlanetLab was used to experiment with 2 scenarios. After the VM placement, some of the physical machines became overloaded while executing the VMs, so to balance the load across physical hosts a VM migration technique was incorporated. The results demonstrate that the average power consumption of the proposed algorithm is less by 27%, SLA violation is reduced by 16% and execution time is decreased by 4% compared to other algorithms. Resource utilisation of the proposed method is increased by 17% as compared to PSO and PADEF algorithms. As shown in the result section, the proposed model outperforms the existing algorithm in underloaded and overloaded conditions. The proposed algorithm succeeded in efficiently placing VMs on hosts in data centres and minimising power consumption, exaction time, SLA violation and the number of VM migrations. The proposed algorithm also increases resource utilisation. In the future, the work will be extended to improve cost efficiency and scalability in the cloud. As an extension of this work, HHO will be merged with machine learning models to achieve better performance and consider additional constraints, such as security and reliability, in cloud computing environments.
